# Intralesional Infiltrations of Cell-Free Filtrates Derived from Human Diabetic Tissues Delay the Healing Process and Recreate Diabetes Histopathological Changes in Healthy Rats

**DOI:** 10.3389/fcdhc.2021.617741

**Published:** 2021-03-26

**Authors:** Jorge Berlanga-Acosta, Maday Fernández-Mayola, Yssel Mendoza-Marí, Ariana García-Ojalvo, Raymond J. Playford, Gerardo Guillen-Nieto

**Affiliations:** ^1^ Tissue Repair, Wound Healing and Cytoprotection Research Group, Biomedical Research Direction, Center for Genetic Engineering and Biotechnology, Playa, Cuba; ^2^ Centre for Immunobiology, Blizard Institute, Barts and The London School of Medicine, Queen Mary University of London, London, United Kingdom

**Keywords:** diabetic foot ulcer, metabolic memory, diabetes, angiopathy (micro- or macroangiopathy), diabetic complication

## Abstract

Lower limb ulcers in type-2 diabetic patients are a frequent complication that tributes to amputation and reduces survival. We hypothesized that diabetic healing impairment and other histopathologic hallmarks are mediated by a T2DM-induced tissue priming/metabolic memory that can be transferred from humans to healthy recipient animals and consequently reproduce diabetic donor’s phenotypes. We examined the effect of human T2DM tissue homogenates injected into non-diabetic rat excisional wounds. Fresh granulation tissue, popliteal artery, and peroneal nerve of patients with T2DM were obtained following amputation. Post-mammoplasty granulation and post-traumatic amputation-tissue of normal subjects acted as controls. The homogenates were intralesionally injected for 6–7 days into rats’ excisional thickness wounds. Infiltration with the different homogenates caused impaired wound closure, inflammation, nerve degeneration, and arterial thickening (all P < 0.01 *vs* relevant control) resembling histopathology of diabetic donor tissues. Control materials caused marginal inflammation only. Infiltration with glycated bovine albumin provoked inflammation and wound healing delay but did not induce arterial thickening. The reproduction of human diabetic traits in healthy recipient animals through a tissue homogenate support the notion on the existence of tissue metabolic memory-associated and transmissible factors, involved in the pathogenesis of diabetic complications. These may have futuristic clinical implications for medical interventions.

## Highlights

The tissue healing response is progressively undermined as a diabetic complication, leading to ulceration, amputation, and mortality. Metabolic memory is invoked as a molecular driver behind the perpetuation of diabetic histopathological hallmarks and clinical complications. This study describes unprecedented evidences indicating that the diabetic impaired healing, as the archetypical peripheral angiopathy and neuropathy, may be faithfully reproduced in healthy animals, through the administration of a cells-free filtrate from diabetic donors’ tissues, with no inter-species barriers. This suggests the existence of transmissible signaling factors, beyond glucotoxicity, involved in the pathogenesis of diabetic damages. These factors seem to impose their message in a short temporary window which incites to reconsider classic concepts of diabetes pathology. The identification of these “drivers” will introduce novel preventive and therapeutic avenues for diabetes.

## Introduction

Diabetes mellitus (DM) is a heterogeneous group of chronic metabolic conditions of pandemic magnitude, characterized by elevated blood glucose levels, resulting from the inability to produce insulin, resistance to insulin action, or both (1).

A torpid healing process along with chronic ulceration and ulcer recurrence constitute a single pathogenic unit and a frequent diabetic complication, which have rendered alarming figures of lower extremity amputations along the history (2). Chronic wounds in general and diabetic foot ulcers (DFU) remain as a persistent medical problem, a scientific challenge, and a socio-economic burden (3). The epidemiological association between an excessive mortality rate with the onset of the diabetic foot syndrome has been documented (2). Accordingly, preventing diabetics’ ulceration, improving their healing response, and prolonging ulcer remission time represent years of survival for this population (4).

Regardless of the pathogenic differences between the two main clinical forms of diabetes, they both share systemic vascular and nerves damages (5). The histopathological hallmarks of diabetic skin angiopathy include perivascular inflammation and collagenization, arterial wall thickening; media layer fibrohyaline degeneration, luminal obliteration, and ultimately vascular rarefaction (6, 7). Peripheral diabetic neuropathy is predominantly characterized by different degrees of nerve fascicles edema, Wallerian degeneration, and myelin fragmentation (8, 9).

The molecular mechanisms underlying diabetic torpid healing and wound chronicity remain elusive. Compelling findings support the existence of a diabetic metabolic memory as a main driver for the perpetuation of multi-organ complications (10–12), including the torpid wound healing response (13, 14).

Recent evidences attest the substantial pathogenic contribution of epigenetic forces for metabolic memory expression, penetrance, and particularly for its generational transmission (15–18). Different evidences converge to indicate that “vascular glycemic memory” (19, 20), and other diabetic complications-related histological hallmarks are promoted by underlying abnormal epigenetic programs (21–23). The fact that various epigenetic mechanisms mediate the intra and transgenerational intra-species inheritance of diabetic-related traits (24, 25), render explanation for our earlier observation that diabetic granulation tissue “inherits,” and morphologically recreates in a period of days (26) the structural changes of chronic evolution which distinctively characterize diabetic skin histology (27–29).

By a series of sequential experiments, this study describes unprecedented evidences indicating that the diabetic impaired healing phenotype, as abnormal histological hallmarks in vessels and nerves, may be reproduced in healthy recipient animals through the administration of different diabetic human donor-derived tissue homogenates. These findings suggest that the molecular “operators” underlying diabetic tissue phenotypes are capable of inter-species transmission that may impose their signature, and ultimately promote host’s tissue remodeling in a diabetic donor’s similar fashion.

## Materials and Methods

### Ethics and Consents

Use of human tissue was approved by relevant local and national regulatory authorities. Subjects gave verbal and informed written consent for the use of material that had been excised for clinical reasons. Diabetic donor materials (granulation tissue and amputated lower limb nerve and artery) were obtained from the National Institute of Angiology and Vascular Surgery (Diabetic Angiopathy Service). The brachial artery from a healthy donor was obtained at the Frank Pais National Orthopaedic Hospital, whereas the control granulation tissue from a healthy donor was collected at the Hermanos Ameijeiras Hospital (Plastic and Reconstructive Surgery Service); all in the city of Havana, Cuba. All animal experiments were approved by local animal ethics committees (Animal Welfare Committee of the Center for Genetic Engineering and Biotechnology, Havana, Cuba). Prevention of pain and distress in our experimental animals meets humane and methodological requisites, since animal suffering impairs the healing process (30, 31).

### Collection and Processing of Samples

#### Collection of Human Samples

Granulation tissue, popliteal artery, and peroneal nerve fragments from a 67 years old male Type-2DM patient afflicted by critical limb ischemia were obtained by dissection immediately after lower limb amputation. This patient underwent major lower extremity amputation due to the critical limb ischemia and the ensued unbearable pain at rest. The lateral side of the distal portion of the right limb was mostly covered by a drought ischemic plaque with no clinical phlogistic changes suggestive of infection. The patient underwent a prophylactic antibiotic scheme 72 h before amputation (flucloxacillin/vancomycin/metronidazole). Recorded Rx reports ruled out signs of osteomyelitis and/or soft tissue infection. Accordingly, this patient was selected as the potential donor because in addition to be clinically non-infected, he was not under the intralesional use of rh-EGF (Heberprot-P), or bone marrow stem cell administration nor any other interventional protocol. Histology of the popliteal artery showed typical changes of peripheral vascular disease comprising intimal proliferation and media thickening with calcified plaques and fatty streaks whereas peroneal nerve tissue demonstrated typical Wallerian degeneration, myelin retraction, and fascicular cavitation. Control granulation tissue was obtained from a 42-year-old healthy female donor undergoing second intent healing by suture dehiscence following cosmetic mammoplasty. Fresh brachial artery tissue was collected from a male healthy donor (45 years old), following surgical amputation due to traffic accident and appeared histologically normal. Immediately following resection/amputation in the surgical room, collected materials were washed with sterile ice-cold normal saline to remove fibrin, blood, and debris. Multiple popliteal artery fragments including deep adjacent soft tissues were 10% buffered formalin fixed for histological analysis and characterization. Other matched fragments were cryopreserved in liquid nitrogen until processing. The target artery and adjacent soft tissue samples were examined on the bases of broadly accepted morphological parameters for arterial vascular pathology (32).

#### Cells-Free Filtrate (CFF) Preparation

Collected tissue was allowed to thaw, weighed and approx. 100 mg of wet tissue placed in 2 ml vial containing 1 ml of normal saline, homogenized using a Tissue Lyser II for 3 min at 30 revolutions per second. Samples were then centrifuged at 10,000 rpm for 10 min at 4°C, sterilized by filtration through 0.2 µm nitrocellulose filters (Sartorius Lab Instruments), aliquoted into sterile Eppendorf vials and stored at −70°C. Prior to use, total protein, glucose concentrations, and cytokine content of the samples were determined using the Bicinchoninic Acid Protein Assay Kit (Sigma-Aldrich, USA) and standard commercial kits for glucose, malondialdehyde (MDA), IL-1β, and IL-6 (all from Abcam, USA). A high sensitivity and specificity ELISA system to measure AGE concentrations was purchased from Wuxi Donglin Sci & Tech Development Co, LTD., China. According to the manufacturer, this kit is endowed with a detection range from less than 33.8 to 8,000 ng/ml (A3-South, 100 # Shuigoutou, Renminxi Rd, Wuxi, Jiangsu, 214031, PRC). Manufacturer’s instructions were followed for the determinations.

### Production of Glycated BSA

Glycated BSA was prepared according to published methods (33). The BSA molecular mass increase and glycation sites were investigated by ESI-MS/MS as previously described to identify AGE formation (34). Sites of glycation using ESI-MS/MS showed glycation of lysine residues 14, 206, 223, 234, 415, 473, 476, 526, 546 (gamm.biocomp.cigb.edu.cu/data/servicios/BSA glucosilada).

### Induction of Skin Wound in Rats, Infiltration With Test Solutions, and Subsequent Analyses

Adult male Sprague Dawley rats (n = 8 per group) were individually housed for 10 days acclimatization in steel grid-bottomed cages (to prevent contamination of wounds with bedding material) and allowed access to standard rodent chow and water *ad libitum* throughout the study. Following acclimatization and under anesthesia [ketamine (80 mg/kg)/xylazine 2% (10 mg/kg) cocktail] each rat underwent two dorsal, symmetrical, retro-scapular full-thickness wounds including *panniuclus carnosum* with a 6 mm diameter disposable biotomes (Acu-Punch, Acuderm Inc., USA) as described (35). Immediately following induction of wounds, animals received local infiltration of test product (100 µg protein in 500 µl saline, or saline alone) into the wound and on a once daily basis until end of experiment. For the initial experiment (granulation tissue injection), animals were sacrificed on day 7 under terminal anesthesia. For experiments 2–4, the protocol was modified to euthanize animals on day 6 to ensure that no wounds were completely closed at the end of the test period, should any homogenate may cause increased healing. Wound size on days 0, 3, 5, and 6 was determined by tracing wound margins on transparent polypropylene films, digitized, and two-dimensional digital planimetric calculation of wound closure determined. Results on day 0 (28 mm^2^) were defined as 100% (36, 37). At the end of the study, animals were killed under terminal anesthesia, the entire wound area with intact surrounding skin excised, fixed in 10% buffered formalin, paraffin-embedded, and sections stained using H&E. Images were captured using a BX43 Olympus microscope, coupled to a digital camera and central command unit (Olympus Dp-21), and processed using ImageJ software (ImageJ 1.48v, NIH, Bethesda, MD, USA) (38). All histological assessments were performed by two independent subjects and an external pathologist under blinded conditions. Inflammatory infiltrate scoring (scored from 0 to 8), and fibro-vascular reaction (based on the degree of collagen bundle formation) were quantitated according to published methods (37, 39). The degree of vascular wall remodeling was quantitated from arterial wall-to-lumen ratios following described procedures (40, 41) and percentage of damaged nerve fibers and degree of Wallerian degeneration determined as described (42, 43).

### Histochemistry and Immunohistochemistry

Tissue sections of human origin and the matched recipient rats were stained with Congo red, and Mallory’s trichrome techniques for amyloid and collagen deposits identification as described (44). Other sections were mounted on poly-l-lysine coated slides (DAKO, Carpinteria, CA, USA) in order to reduce inter-tissue/experimental variations during immunohistochemistry studies. The slides corresponding to three wounds per sub-group (except for protocol 3) were dewaxed and rehydrated through graded washes of ethanol. Rehydrated slides were exposed to antigen retrieval solution for 20 min at 80°C and washed with 0.05 M tris-buffered saline (pH 7.6) for 5 min. Endogenous peroxidase was blocked, and subsequently the tissue sections were exposed to pre-heated antigen retrieval solution high pH (DAKO) for 10 min. Following equilibration to room temperature non-specific binding blocking solution was used for 20 min. The sections were then incubated for 40 min with antibodies directed to: (1) TNF-α (Ab6671), (2) AGE (Ab23722), (3) RAGE (Ab228861), (4) e-NOS with a “gain-of-function” phosphorylation in Ser-1177 (Ab75369), and (5) an active isoform of NFκβ (p65) phosphorylated in serine 529 (Ab97726), according to manufacturer’s specification. The immunolabeling reaction was developed as described for the Mouse and Rabbit Specific HRP/DAB (ABC) Detection IHC kit (Ab64264) and semiquantitatively graded as described (45). As internal immunoreaction reference, a granulation tissue fragment of the donor’s diabetic-ischemic ulcer was used. Previous studies from our group had immunohistologically characterized this type of tissue (46). Non-specific tissue labeling internal controls included the omission/replacement of the primary antibody by the background reducing antibody diluent (Ab6424), and normal rabbit serum (Boster Biological Technology, Pleasanton CA, USA, catalog # AR1010).

### Study Protocols and Experimental Sequence

#### Study 1. Effect of human diabetic ischemic ulcer granulation tissue on rat skin wound healing and histology.

Rationale: To determine if the healing response was impaired and histological changes occurred in rat wounds when injected with T2DM ischemic chronic ulcer-granulation tissue CFF, as compared to human healthy granulation tissue control.

Three groups of rats (N = 8 per group with two wounds per rat, giving 16 wounds per condition) received wound infiltration of:

1. Saline alone (control)
2. Tissue CFF from “normal” granulation tissue from a subject that had undergone breast cosmetic surgery
3. Tissue CFF from granulation tissue derived from T2DM chronic ulcer

#### Study 2. Effect of human diabetic arterial tissue CFF on rat skin wound healing and histology.

Rationale: To examine if arterial tissue distant from ulcerated areas of T2DM patient caused similar healing impairment and angiopathy changes to those seen in experiment 1.

Three groups of rats (N = 8 per group with two wounds per rat, giving 16 wounds per condition) received wound infiltration of:

1. Saline alone
2. Brachial artery CFF from a non-diabetic healthy donor (control group)
3. Popliteal artery-derived CFF from T2DM patient

#### Study 3. Effect of T2DM human nerve CFF on rat skin wound healing and histology.

Rationale: To examine if nerve tissue distant from ulcerated areas of T2DM patient caused similar histopathological changes to those described for experiments 1 and 2, including nerve fascicles degeneration.

Two groups of rats (N = 8 per group with two wounds per rat, giving 16 wounds per condition) received wound infiltration of:

Rats received wound infiltration of

1. Saline alone
2. Peroneal nerve-derived CFF from same T2DM patient as studies 1 and 2

#### Study 4. Relevance of protein glycation on rat wound healing and histology.

Rationale: To examine if injection with a glycated protein (BSA) caused healing impairment and similar histopathological changes to those described in experiments 1–3 when diabetic tissues CFFs were used.

Three groups of rats (N = 8 per group with two wounds per rat, giving 16 wounds per condition) received wound infiltration of:

1. Saline alone
2. Native BSA
3. Glycated BSA

### Statistical Analyses

Statistical analyses were performed using GraphPad Prism software 6.01 (La Jolla, CA, USA). For analyses comprising more than two groups, the Kruskal-Wallis test was performed followed by Dunn’s multiple comparisons test. Comparisons between two groups were analyzed using the Mann Whitney test. p-value <0.05 was considered statistically significant. All results are expressed as mean ± SD.

## Results

### Study 1. Effect of Human Diabetic Ischemic Ulcer Granulation Tissue on Rat Wound Healing Response and Local Histology

Diabetic ischemic ulcer granulation tissue exhibited the largest concentrations of MDA, IL-6, and AGEs as compared to the relevant healthy donor-control granulation tissue. As shown in **Table 1**, diabetic homogenate levels of MDA and IL-6 levels largely exceeded the rest of the samples.

**Table 1:** Descriptive characterization of the experimental samples.

Samples	MDA/mg protein (nmol/mg)	IL-6/mg protein (pg/mg)	IL-1β/mg protein (pg/mg)	AGE/mg protein (ng/mg)
Diabetic ischemic ulcer granulation tissue	1.363	44.84	32.40	2.966
Healthy donor-granulation tissue	0.836	0.64	14.13	0.260
Artery T2DM	0.275	16.67	50.58	2.615
Artery-healthy donor	0.601	10.43	16.97	0.676
Nerve T2DM	0.192	0.39	26.02	0.631
Glycated BSA	–	–	–	683.8

**Figure 1:**
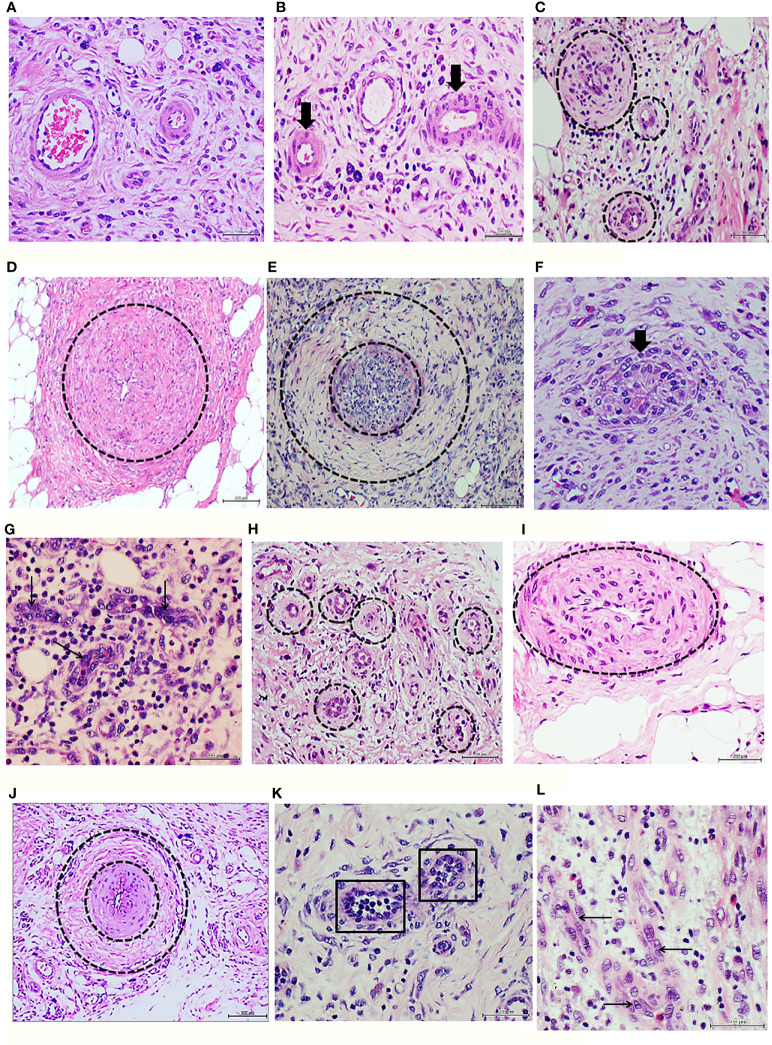
Panels 1A to 1G correspond to protocol 1. Characterization of the ischemic diabetic granulation tissue donor. **(A)** Histological image of human normal granulation tissue sample obtained from debridement upon a hypertrophic granulative response. The presence of normal caliber vessels, minimal infiltration of round mononuclear cells, and woven collagen eosinophilic bundles is shown. Healthy female donor, cosmetic mammoplasty-derived sample. H/E. Magnification ×20. **(B)** Image representative of a granulation tissue of rats injected with healthy donor, human normal granulation tissue. Note the normal morphology and the luminal aspect of two arterioles (arrows). Normal infiltration of round basophilic inflammatory cells embedded in an incipient collagen fibers matrix are observable. H/E. Magnification ×20. **(C)** Histological image of the diabetic ischemic ulcer granulation tissue. It is evident the presence of arteriolar wall thickening (circles) since very early stages of the angiogenic response. A dense fibrotic matrix infiltrated with inflammatory cells is noticed. H/E. Magnification ×20. **(D)** Representative image of arteriolar wall thickening through media and intima layers engorgement and fusion (circle) with luminal narrowing in the granulation tissue of the ischemic diabetic ulcer used to prepare the homogenates for protocol 1. H/E. Magnification ×20. **(E)** Exaggerated concentric fibrogenic accumulation within the media layer sector. The inner encirclement relates to the intima layer and an intraluminal cellular infiltration—possibly upon a thrombus recanalization. The outer circle delimits the collagen accumulation. H/E. Magnification ×20. **(F)** Endothelial collar hyperplasia with bulky cells aspect and disorientation (arrow). It is evident the vertical orientation of the cells targeting or encroaching to the luminal side. H/E. Magnification ×40. **(G)** Representative of an abnormal process of venular organization. The arrows indicate to the giant aspect of the endothelial nuclei and the abnormal cellular co-opting leading to luminal obliteration. H/E. Magnification ×40. Panels 1H to 1L correspond to protocol 1. Characterization of the granulation tissue of rats treated with the ischemic diabetic granulation tissue. **(H)** Representative angiogenic response showing precocious arteriolar wall thickening (circles) similar to that observed in the human diabetic donor. H/E. Magnification ×40. **(I)** Rats arteriolar wall thickening with consequent luminal collapse in which intima and media layers appear hypertrophic and fused. H/E. Magnification ×40. **(J)** Image representing two pathologic changes observed in the rats treated with the diabetic granulation tissue: intima and media layers hypertrophy and fusion (internal circle) with luminal narrowing and concentric adventitial collagen accumulation (outer circle). H/E. Magnification ×20. **(K)** The squares delimit two incipient arterioles in the granulation tissue of rats treated with the cognate diabetic human material in which the endothelial collar exhibits cellular hyperplasia and disorientation. H/E. Magnification ×40. **(L)** The abnormal venular organization observed in the diabetic granulation tissue donor sample is reproduced in the recipient rats. Again, the arrows point to giant endothelial cells with an abnormal co-opting and no luminal aspect. H/E. Magnification ×40.

**Figure 2:**
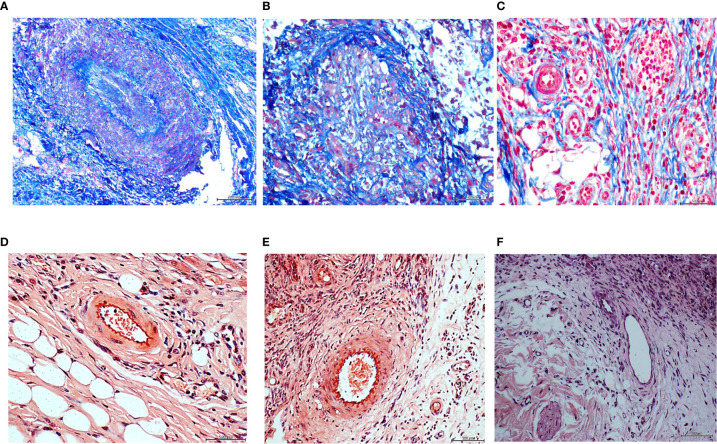
Panels **(A–C)**. Mallory trichrome reaction for collagen identification. Intense positive reaction to Mallory staining corresponding to a vascular structure in the ischemic ulcer granulation tissue of the diabetic donor **(A)**. Collagen (blue stain) involves the walls and encroaches into the lumen leading to complete obliteration. Periadventitial collagen fibers are also identifiable. Rats treated with the ischemic ulcer granulation tissue- **(B)**, also showed a collagen deposit disorder leading to luminal encroachment of Mallory stained bundles. No perivascular, intramural, or intraluminal collagen accumulation was detected in the granulation tissue samples of the rats receiving the homogenate of the healthy donor/normal granulation tissue **(C)**. Congo red, suggesting a possible amyloid material accumulation in the endothelial collar of vessels and adjacent area is shown in panels D and E for the diabetic ulcer granulation tissue donor, and the recipient rats, respectively. No positive reaction to Congo red **(F)** was detected in the samples derived from healthy donor/normal granulation tissue –treated rats.

**Figure 3:**
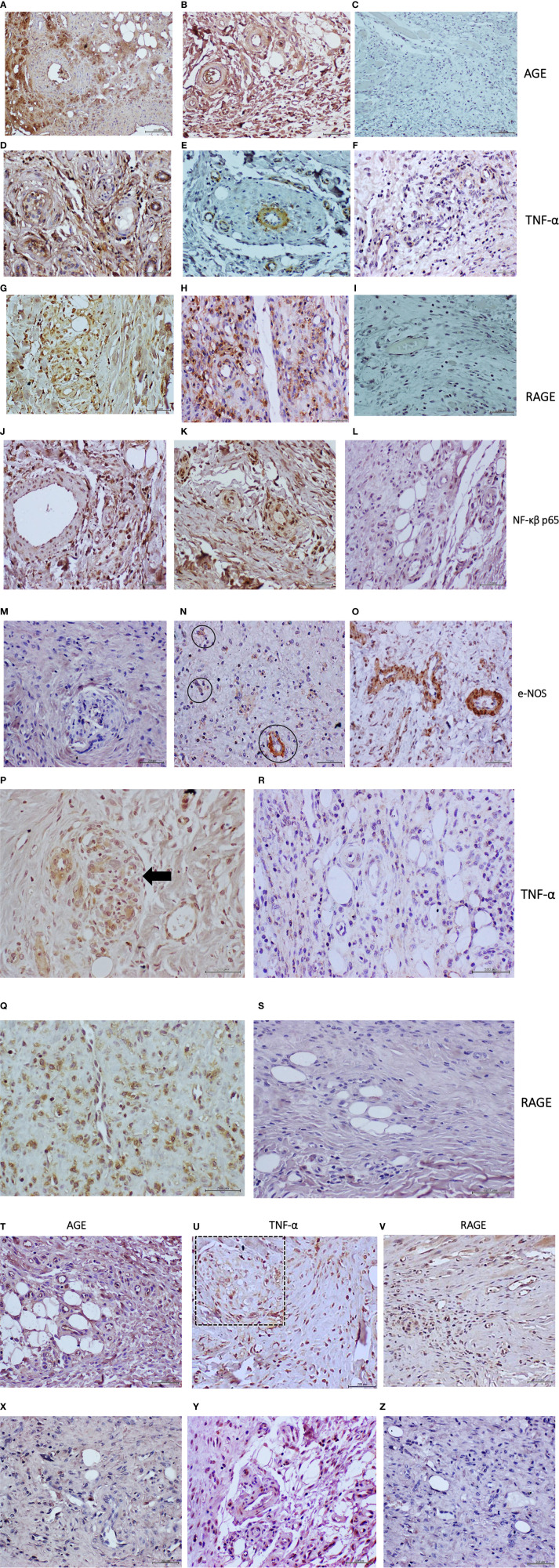
Immunohistological characterization of rats’ granulation tissue to the administration of T2DM or healthy-normal tissue cells-free filtrates. Panels from **(A–O)** corresponding to protocol 1. Diabetic ischemic ulcer granulation tissue CFF administration. Panels **(A–C)**, correspond to AGE expression in diabetic granulation tissue donor sample, the matched recipient rats, and rats treated with healthy donor granulation tissue, respectively. **(A)** Intense labeling of AGE accumulation in the granulation tissue extracellular matrix as in the walls of a pathologic arteriole. **(B)** AGE is also largely accumulated in the granulation tissue and microvascular structures, including the endothelial layer of rats receiving the diabetic granulation tissue homogenate. **(C)** No AGE expression is detected in the animals treated with the healthy donor granulation tissue from a cosmetic mammoplasty. TNF-α expression is shown in panels **(D–F)**. As shown by panel **(D)**, this cytokine is intensely expressed by different granulation tissue structures, including the vascular collar. The rats treated with the diabetic material likewise show TNF-α expression in the endothelial collar. A thickened arteriole, along with other adjacent micro arterioles is shown in panel **(E)**. Rats treated with the healthy donor’s granulation tissue homogenate also express TNF-α in granulation tissue cells but in a well-delimited pattern and with far less intensity, panel **(F)**. RAGE is abundantly expressed by granulation tissue infiltrating cells within the diabetic donor granulation tissue as by few cells embedded in the vascular collar—panel **(G)**. A similar pattern and intensity of expression are observed in the diabetic material recipient rats. As shown in panel **(H)**, cells of the microarterioles collar are clearly positive to RAGE expression. RAGE is not expressed by the rats treated with the healthy donor’s granulation tissue homogenate – panel **(I)**. The descried inflammatory profile is in line with the findings obtained with an antibody targeting an active NF-κβ p65. For both, the granulation tissue sample of the diabetic donor panel **(J)** and for the recipient rats panel **(K)**, p65 is detected mostly in the nucleus of a multitude of cells, including the vascular wall and the endothelial layer. In contrast, p65 is faintly expressed by some cells and incipient microvascular structures within the granulation tissue of control rats, treated with the healthy donor homogenate —panel **(L)**. e-NOS expression is almost absent in the vascular structures of the diabetic donor ischemic granulation tissue—panel **(M)**. This derangement is also detected in the granulation tissue vessels of the diabetic material recipient rats. As shown in panel **(N)**, few vascular structures (encircled) express e-NOS, and importantly, this expression seems to decline when the morphology of the venules is abnormal. Panel **(O)** demonstrates the intense expression of e-NOS by the walls of morphologically normal vessels in rats treated with the healthy donor granulation tissue homogenate. Panels **(P–S)** corresponding to protocol 2. Diabetic PAD arterial tissue CFF administration. As shown in panels **(P, Q)**, the administration of the diabetic arterial homogenate triggered an inflammatory response given by a neat expression of TNF-α and RAGE, respectively, in the granulation tissue of otherwise normal recipient rats. The former was mostly circumscribed to vascular structures. Panel **(P)** shows a nest of de novo formed microarterioles (arrow) in which TNF-α is detected. RAGE shows a broader expression in cells infiltrating the granulation tissue panel **(Q)**. In sharp contrast, TNF-α is limitedly and marginally expressed by some cells within the microscopic field, whereas RAGE was not expressed by the components of the granulation tissue of the rats treated with the healthy donor material (panels R, S, respectively). Panels **(T–Z)** corresponding to protocol 4. Effect of glycated BSA administration. As illustrated in panels **(T–U)**, and in contrast to the effect of native BSA, the administration of glycated BSA promoted the accumulation of AGE in the extracellular matrix, as in most microvascular structures including the endothelial lining panel **(T)**. Accordingly, TNF-α was detected in a broad constellation of cells, microvessels walls, as in a structure similar to a nerve fascicle (Panel **U,** square). RAGE is also immunodetected in infiltrating cells as in the endothelial collar of vascular structures panel **(V)**. Native BSA treated rats did not show to accumulate AGE in any structure/cell of the granulation tissue panel **(X)**. As described formerly, TNF-α expression is marginal and limited to some cells and incipient microvascular structures panel **(Y)**. Native BSA did not show to induce RAGE expression panel **(Z)**. All microphotographs magnification was x 40. Mayer’s hematoxylin counterstain.

**Figure 4:**
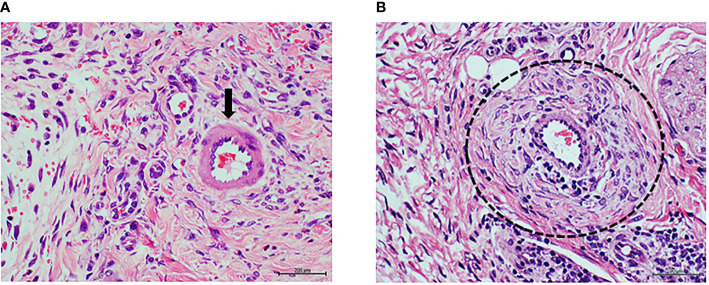
Panels **(A**, **B)** correspond to protocol 2. Granulation tissue of rats treated with arterial tissue homogenate. **(A)** Image representative of the histological response of the granulation tissue of rats treated with healthy donor/normal artery in which tissue and vascular architecture is normal. The arrows indicate to an arteriole with normal walls and patent luminal aspect. Abundant number of fusiform, fibroblasts-like cells in a woven collagen matrix is observed. H/E. Magnification ×20. **(B)** Representative image of the vascular response in rats treated with the diabetic artery homogenate. The circle targets an artery with a remarkable wall thickening at the media layer sector with hypercellularity in which fusiform cells are overrepresented. A conglomerate of round, basophilic mononuclear cells is also observable about the lower right corner. H/E. Magnification ×40.

**Figure 5:**
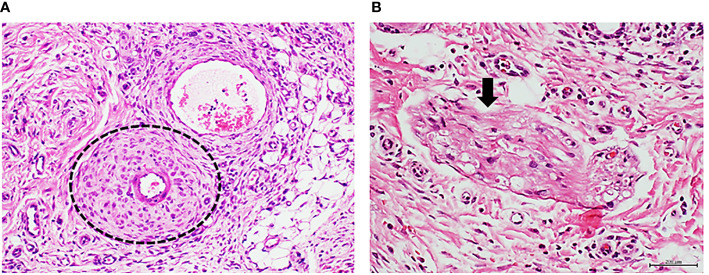
Panels **(A**, **B)** correspond to protocol 3. Granulation tissue of rats treated with diabetic peripheral nerve tissue homogenate. **(A)** Representative image of the vascular response of rats treated with a diabetic nervous tissue homogenate. As described for the diabetic artery response—here again, there is a clear arteriolar wall thickening at the media layer sector with hypercellularity in which some fusiform-like cells are concentrically aligned at the periphery. H/E. Magnification ×20. **(B)** The arrow points to the section of a nerve fascicle in which degenerative changes including demyelination, contraction, and cavitation are associated to the administration of the diabetic nerve tissue homogenate. H/E. Magnification ×40.

Infiltration with human healthy donor granulation tissue homogenate did not delay wound healing, and did not cause morphological changes when compared against the effect of saline injections (**Table 2**). Histology of the initial human granulation tissue from healthy donor (**Figure 1A**) and the corresponding rat wounds injected with either saline or healthy granulation tissue (**Figure 1B**), all showed a mild infiltration with lymphocytes, normal caliber vessels, and well organized/mature collagen fibers. In contrast, infiltration with granulation tissue from the T2DM subject resulted in delayed wound healing, a marked inflammatory infiltrate mainly based on lymphocytes, and a constellation of abnormal vascular histological changes that mirrored those of the human donor T2DM granulation tissue. The diabetic donor’s granulation tissue angiogenic response was mostly abnormal and incomplete. According to the predominant pathological changes we describe it as: (1) precocious vascular walls thickening since early angiogenic sprouting stage (**Figure 1C**), (2) arteriolar walls thickening with luminal narrowing on the bases of media layer expansion and invasion into the intima with layers fusion (**Figure 1D**), (3) exaggerated media-concentric collagenization (**Figure 1E**), (4) endothelial cells collar hyperplasia, or with a bulky aspect, or onset of a fusiform, fibroblastic-like phenotype that project into the lumen (**Figure 1F**), (5) abnormal or incomplete venular organization (**Figure 1G**).

**Table 2:** Effect of infiltration with cells-free homogenates obtained from T2DM patient, control (non-DM) subject tissues, or glycated bovine serum albumin (BSA).

Study	Infiltrate constituent	Wound closure (%)	Arterial thickening (wall-to-lumen ratio)	Nerve fibers damaged (%)	Inflammationscore
1. Granulation tissue homogenate	T2DM skin ulcer granulation tissue	76.63 ± 8.48^*§^	1.86 ± 0.60^**§§^	89.24 ± 12.03^**§§^	7.79 ± 1.92^**§§^
	Cosmetic mammoplasty granulation tissue	87.08 ± 5.63	0.33 ± 0.13	49.41 ± 17.54	4.67 ± 2.04
	Saline	87.47 ± 8.04	0.38 ± 0.14	40.97 ± 24.09	2.08 ± 0.28
2. Arterial homogenate	Popliteal artery from T2DM patient	34.73 ± 13.91^**§§^	1.78 ± 0.55^**§§^	84.40 ± 12.00^**§§^	7.88 ± 1.86^*§§^
	Healthy artery	53.78 ± 13.30	0.37 ± 0.12	53.47 ± 29.35	5.57 ± 2.39
	Saline	52.45 ± 9.55	0.34 ± 0.11	40.80 ± 15.94	2.03 ± 0.17
3. Nerve homogenate	Peroneal nerve from T2DM patient	33.14 ± 21.04^§§^	1.15 ± 0.70^§§^	59.31 ± 19.25^§§^	6.94 ± 2.43^§§^
	Saline	57.60 ± 10.25	0.34 ± 0.09	39.41 ± 18.13	2.19 ± 0.40
4. Glycated bovine serum albumin	Glycated BSA	29.77 ± 17.76^**§§^	0.37 ± 0.13	79.25 ± 15.63^**§§^	7.98 ± 2.17^**§§^
	Native BSA	41.96 ± 8.99	0.36 ± 0.11	41.06 ± 17.13	4.36 ± 2.12
	Saline	48.61 ± 10.59	0.35 ± 0.11	40.95 ± 20.53	2.05 ± 0.22

Healthy, non-diabetic rats had standard wounds inflicted on day 0 and received once daily intralesional infiltrations with cells-free homogenates or BSA containing 100 ug protein in 500 ul saline, or saline alone. Animals in study 1 were terminated on day 7 whereas animals in studies 2–4 were autopsied on day 6. Wound size on day 1 was defined as 100%. Inflammatory infiltrate scoring (scored 0–8), fibro-vascular reaction, arterial wall-to-lumen ratios, and degree of Wallerian degeneration assessed according to published methods (see Materials and Methods). * and ** indicates P < 0.05 or <0.01 vs saline, § and §§ indicates P < 0.05 or <0.01 vs tissue or BSA controls.

The recipient rats treated with the diabetic granulation tissue-derived homogenate reproduced in their granulation tissue most of the above described abnormalities. Accordingly, this heterogeneous group of changes includes: (1) vascular walls thickening since early stages (**Figure 1H**), (2) arteriolar walls thickening with luminal narrowing (**Figure 1I**), (3) exaggerated periadventitial concentric collagenization (**Figure 1J**), (4) endothelial collar hyperplasia with bulky aspect of some cells (**Figure 1K**), (5) abnormal or incomplete venular organization (**Figure 1L**). Again, none of the above described changes were detected in the rats treated with the healthy donor/normal artery recipient rats (**Figure 1B**).

In order to further characterize these unprecedented findings we conducted histochemical and immunohistochemical reactions, in which the diabetic donor granulation tissue and the matched recipient rats’ were compared, concurrently having the healthy arterial donor recipient rats’ as controls. The formerly described arteriolar fibrogenesis in both the diabetic donor (**Figure 2A**) and the matched recipient rats (**Figure 2B**) proved to be positive to Mallory trichrome reaction. Furthermore, similar to the diabetic donor tissue sample (**Figure 2D**), the vascular walls of the recipient animals’ granulation tissue were positive to Congo red reaction (**Figure 2E**). The control rats treated with the healthy donor homogenate were positive to collagen in a physiological pattern and convincingly negative to Congo red, neglecting any amyloid material vascular accumulation (**Figures 2C, F**).

The human diabetic tissue sample exhibited intense AGE reactivity which was mostly accumulated in the granulation tissue extracellular matrix in proximity to vascular structures (**Figure 3A**). Correspondingly, AGE reactivity was also identified in the endothelial collar as in granulation tissue matrix of the diabetic material recipient rats (**Figure 3B**). No AGE accumulation was evidenced in the granulation matrix of control rats receiving the human healthy donor granulation tissue homogenate (**Figure 3C**). Intense RAGE expression was observed in vascular walls and adjacent cells in both the diabetic donor and the cognate recipient rats’ granulation tissue (**Figures 3D, E**); whereas this marker was not detected in the control rats (**Figure 3F**). TNF-α expression was intensely detected in granulation tissue infiltrating cells and in vascular structures cells of both the diabetic donor and the recipient rats’ tissue (**Figures 3G, H** respectively). Very limited expression was identified however in round cells scattered across the granulation tissue field of control rats treated with the healthy donor material (**Figure 3I**). Probing the pathologic donor (**Figure 3J**) and the recipient rats (**Figure 3K**) samples with an antibody generated against an active isoform of NF-κB, showed a p65 intense expression in round, possibly inflammatory cells, as in other cells integrated to the arteriolar walls. In contrast, a pale and very well-circumscribed p65 signal was found in a limited number of cells in the healthy donor recipient control animals (**Figure 3L**). In correspondence to the described vessels structural damages, the e-NOS expression appeared deficitary in the diabetic donor tissue (**Figure 3M**), as in the matched recipient rats. Interestingly, e-NOS expression intensity and distribution seemed to parallel the vascular damages (**Figure 3N**). This is substantially contrasting with the e-NOS pattern of expression in the vessels walls of the healthy donor recipient rats (**Figure 3O**). Finally, formal histological scoring based on wall-to-lumen ratio confirmed the findings of arterial thickening and luminal obliteration, which was associated to nerve damage, and inflammatory scores in the T2DM treated wounds (**Table 2**).

### Study 2. Effect of Human Diabetic Arterial Tissue Homogenate on Rat Wound Healing Response and Local Histology

In sharp contrast to the values detected in the homogenate derived from the healthy donor artery, the homogenate obtained from the ischemic diabetic counterpart contained the highest levels of both IL-1β and IL-6 with considerable AGEs accumulation (**Table 1**).

The rate of wound healing was similar in saline-treated or normal arterial tissue-treated animals, but significantly delayed in wounds that received arterial tissue homogenate from the ischemic T2DM donor patient (**Table 2**). Histology of rat wounds injected with the normal arterial tissue CFF showed a healthy granulation tissue appearance with normal vessels and no arteriolar remodeling (**Figure 4A**). However, wounds treated with T2DM arterial homogenate had appearances similar to that found when diabetic ulcer granulation tissue was used (Study 1). Changes included abnormal and incomplete angiogenesis, endothelial collar hypertrophy, and media layer sector infiltration by cells and concentric collagenization (**Figure 4B**).

Formal histological scoring confirmed these findings of increased arterial thickening, nerve damage, and inflammatory scores, all being significantly increased (**Table 2**). Immunostaining showed that as compared to normal/healthy artery-derived homogenate, the wounds treated with the diabetic arterial homogenate exhibited intense expression of both TNF-α and RAGE. TNF-α appeared to be far more circumscribed to vascular structures (**Figure 3P**) whereas RAGE immunolabeling was found in a large number of cells within the granulation tissue extracellular matrix (**Figure 3Q**). Arterial healthy donor-treated rats showed a pale TNF-α expression by some cells infiltrating the granulation tissue filed (**Figure 3R**) inasmuch RAGE did not appear to be expressed (**Figure 3S**).

### Study 3. Effect of T2DM Human Nerve Homogenate on Rat Wound Healing Response and Local Histology

Fascicle fragments of the peroneal nerve of the amputated diabetic donor exhibited histopathological evidences of Wallerian degeneration, with intense degree of myelin retraction, lysis, and fascicular cavitation (not shown). Of note, diabetic nerve tissue sample showed the lowest concentration levels of MDA, IL-6, and AGE similar to those detected for healthy donor artery (**Table 1**). Analogous to the effect induced by the diabetic artery homogenate, only 33% of the injured area appeared to be closed in the wounds treated with the diabetic nerve-derived homogenate (**Table 2**). Furthermore, this homogenate also promoted arterial wall thickening, mostly supported by a dramatic hypercellularity and collagenization in the media layer sector (**Figure 5A**). Wallerian degeneration in a severe degree was also promoted by the diabetic nerve material infiltration (**Figure 5B**), likewise reproducing the observations accounted with the two previous diabetic tissue samples.

### Study 4. Relevance of Protein Glycation on Wound Healing Histology

**Figure 6:**
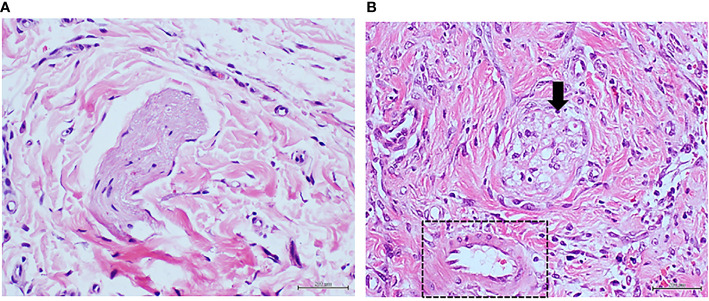
Panels **(A**, **B)** correspond to protocol 4. Granulation tissue of rats treated with highly glycated BSA. **(A)** Image representative of the normal histology of a nerve fascicle and adjacent microvascular, and matrix structure of animals treated with non-glycated, native BSA. It is noticeable the perfect myelin preservation within the endoneurium. H/E. Magnification ×40. **(B)** Highly glycated BSA damaged the nerve fascicles which exhibit severe myelin loss with the subsequent aspect of cavitation (arrow). Of note, however, is the observation that the abnormal hyperglycated environment did not promote any type of arteriolar wall remodeling (square). H/E. Magnification ×40.

The AGEs levels determined in the glycated BSA samples were 683.8 ng/mg of protein, which was 230 times higher than the highest value determined for the diabetic tissue samples (**Table 1**). This highly glycated BSA significantly impaired wound closure rate as compared to saline or normal BSA-treated animals (**Table 2**). Compared to animals receiving saline, those that had received native BSA showed normal histology with no differences in granulation tissue structure, maturation, histological patterns of angiogenesis, inflammatory infiltrate intensity, and nerve fascicles integrity (**Table 2**; **Figure 6A**). Glycated BSA, however, caused significant increase of inflammatory infiltrate and nerve fascicle degeneration consisting in myelin retraction, lysis, and cavitation (**Table 2**). Noteworthy, from the qualitative analysis and the morphometric measurements, arteriolar morphology was not altered by glycated BSA. Wall-to-lumen ratio data were very similar among the three groups in the experiment (**Table 2**). Thus, glycated BSA administration did not result in arteriolar thickening (**Figure 6B**). Further immunohistological examinations of these samples showed that glycated BSA promoted a well-delimited AGE accumulation in the granulation tissue-emerging microvasculature (**Figure 3T**), which was coincident with the increase of TNF-α (**Figure 3U**) and RAGE expression (**Figure 3V**) in granulation tissue structures and infiltrating cells. Of note, granulation tissue samples derived from the rats treated with native BSA did not show AGE accumulation (**Figure 3X**). Again, TNF-α was limitedly expressed by scar area infiltrating cells and microvascular structures (**Figure 3Y**). Finally, RAGE expression was not detected (**Figure 3Z**).

## Discussion

Despite the limitations of this work (absence of a control healthy donor-derived peripheral nerve, limited number of samples, and incomplete mechanistic insights—including lack of studies addressing the VEGF axis), it demonstrates for the first time that the torpid healing phenotype and cutaneous histopathological hallmarks of diabetes can be experimentally reproduced in a laboratory animal species through the injection of CFF obtained from human diabetic tissue samples.

Given the fact that the first experiment of passive transference of diabetic wound granulation tissue homogenate to healthy rats’ wounds, translated in an unexpected phenotypical recreation of the donor’s arteriolar wall thickening and nerve damages; subsequent experiments examined the potential impact of diabetic artery, and nerve tissues homogenates. Accordingly, this experimental series with diabetic tissues homogenates steadily rendered four major reproducible findings: (1) wound closure delay, (2) arteriolar wall thickening: endothelial encroachment/perivascular hypercellularity and collagenization, (3) nerve fascicle degeneration, and (4) intense inflammation; which were never detected when granulation or arterial tissue homogenates from healthy donors were administered. The histological findings presented here faithfully represent the results of early preliminary pilot experiments, which were supporting for this experimental protocol in terms of dose and time frame of administration. It is worth mentioning that the immunohistochemical characterization studies allow to propose that “diabetic memory transference” was not limited to morphological traits, as it appeared to include diabetic donor’s intrinsic functional alterations like the nuclear expression of p65 phosphorylated in serine 529, and the deficient profile of a hyperactive isoform of e-NOS in rats treated with the diabetic granulation tissue homogenate. The drivers behind this “inherited dysfunctionalities” from the human diabetic donor, to rats’ wound neovessels remain to be identified. Nevertheless, the overexpression of phosphorylated p65 in the diabetic material recipient animals is not attributable to a canonical host *versus* graft activation as it was only marginally expressed in those rats receiving the healthy human donor granulation tissue. It is also noteworthy that T2DM tissue caused positive Congo red staining in the endothelial collar of the vessels in the granulation tissue of recipient rats. Whether this finding represents passive transference of toxic amyloid, a similar reactant material, or a proximal signaling factor promoting an acute vascular amyloid accumulation remains unknown. This histochemical marker was not identified in the rats’ samples treated with the healthy donor/normal granulation tissue.

The production of a standardized skin wound in rodents is well established for examining factors that enhance or delay the rate of skin healing (35). The initial studies (granulation tissue experiment) allowed 7 days for wound recovery but was modified to 6 days for subsequent studies to ensure that wounds were not completely closed at harvesting should a test material increase, rather decrease healing. Previous studies examining the effects of diabetes on wound repair used animals that are diabetic through genetic abnormalities (47, 48) or through administration of compounds such as streptozotocin (49, 50). In contrast, our hypothesis was not reliant on manipulating glucose control in the test animals and we therefore used non-diabetic animals. Thus, our demonstrations on the reproduction of this DM-ulcer phenotype were generated on the background of a normal rat with healthy microvascular and peripheral nervous systems.

We considered tissue needed to be collected and processed immediately in the operating room to prevent deterioration. This limited the number of samples that could be obtained and as we were unable to acquire a sample of a “normal” nerve, effects of T2DM nerve tissue on wound healing were compared against a saline control. All tissue homogenates were microfiltered (0.2 µm) to ensure sterility. This does not exclude that microbial by-products were present in the T2DM ulcer homogenate, but not in the control, or T2DM artery or nerve samples, as they were collected under sterile conditions in the operating room. As similar results were seen using all three T2DM tissue sites, bacterial products are unlikely to be cause of the changes observed. Furthermore, the patient was not in a septic status, and the major amputation was decided due to critical limb ischemia (classified as Rutherford’s category 5), intractable ischemic pain, and not by bone or soft tissue invasive infection. Reviewing of the image study reports allowed us to rule out infection in bone and/or deep soft tissues of the target limb.

Our finding that normal human tissues did not cause the pathological abnormalities seen using T2DM tissue also demonstrates that T2DM-type histological and morphological changes were not due to generic introduction of human xenogeneic material, but triggered by aberrant molecular signalers present within the T2DM tissues. This suggests that in the tissue lysate soluble messengers are contained, acting as driving forces toward the torpid wound healing and an abnormal vascular organization.

The membrane RAGE interacts with a diverse range of endogenous ligands generically termed AGEs. AGE formation is markedly increased in serum and tissues, including atherosclerotic plaques of DM patients (51), and in keeping with this, the cell free homogenates derived from the T2DM granulation and arterial tissues had higher MDA, pro-inflammatory cytokines, and AGEs content compared to their relevant controls. Given that there is evidence on the pathogenic role of the AGE/RAGE signaling pathway in diabetes vascular disease (52–54), and having observed increased RAGE and TNF-α expression in rat wounds injected with T2DM tissues in each experimental protocol; we investigated if AGE/RAGE interactions were the common pathway to explain our results. Thus we tested glycated BSA as an exemplar AGE product in our rat wound model (55). Although glycated BSA delayed healing, increased inflammation, induced nerve injury, and RAGE expression, it did not result in arteriolar thickening despite the high concentration of AGEs (683.8 ng/mg) attained in our glycation process. In line with this, the diabetic nerve tissue homogenate proved to promote arterial wall hypercellularity and thickening, despite containing the lowest concentrations of AGEs. These evidences suggest that factor(s) other than the toxic impact of highly glycated proteins and inflammatory program-associated signalers within the homogenate, are responsible for the observed vascular wall remodeling. Primarily, a direct potential glucotoxic effect was ruled out because glucose concentrations in the homogenates were all below 5 mmol/L. Previous studies had shown that delayed wound healing in diabetes is not caused by local high-glucose concentration itself (56). Nevertheless, future studies will examine the presence of intermediate glycation products, and of advanced lipoxidation end-products in the donor tissues homogenates, given their atherogenic nature and their potential contribution to the pathologic vascular remodeling observed in the recipient rats (57, 58).

The factor(s) involved in the phenomenon described here may not be a single molecule, but a combination of signaling agents such as present within cell exosomes. Exosomes that comprise a constellation of subcellular fragments appear to play important roles in cell-to-cell communication, horizontal gene transfer, immune modulation, and participate in the development of diabetes and its associated complications (59). In support of this notion, recent studies suggest that exosomes may play an important role in the pathophysiology of degenerative conditions such as retinal degeneration (60). Further work is required to examine these ideas. The futuristic implications of these evidences anticipate additional studies in search for evidences reproducibility, as this protocol was based on the tissues collected from a single diabetic donor.

Current reviews of the pathogenesis of diabetic foot ulceration describe the contributions of neuropathy, microangiopathy, and impaired metabolism (61). Our studies demonstrated an additional contribution of metabolic memory/tissue priming “drivers” present both within the ulcer and in distant, internal tissues as arteries and nerves. Although we have not identified the exact causative factor within the T2DM tissue responsible for the transmitted changes, our studies have several clinical implications. **A**. Microvascular changes, assumed to be slowly progressive in T2DM patients, can be rapidly transmittable into a non-diabetic rat model, suggesting pathological vascular changes may occur much quicker than considered currently. **B.** The interspecies transmission of a chronic granulation tissue phenotype, even when the recipient animal is not diabetic and has a normal vascular bed, shows donor T2DM ulcers contain local tissue signaling factors that may be involved in ulcer initiation, extension, perpetuation, and relapse. **C**. Delayed healing with associated tissue pathological changes was transmissible using T2DM arterial and nerve tissue, distant from the actual ulcer area, suggesting metabolic memory/tissue priming is present in T2DM tissues distant from ulcerated areas **D.** Generalized pro-ulcerogenic tissue priming, independent of vascular insufficiency, may help explain why even mild degrees of trauma can precipitate serious ulceration in T2DM patients.

Taken together, our studies suggest that, in addition to the well-established risk factors of vascular insufficiency, neuropathy, and trauma, tissue priming/metabolic memory may be involved in the pathogenesis, failure to heal, and frequent recurrence of skin ulceration seen in T2DM patients. Furthermore, the drivers of these tissue priming/metabolic memory-derived events can be transferred from diabetic humans to normal healthy animals, excluding the existence of inter-species barriers and imposing their diabetic histologic archetypical damages program.

### Clinical Perspectives

- Lower extremity ulcers in diabetic patients are recalcitrant to heal and frequently recur. We considered T2DM might induce changes in tissue signaling factors (tissue priming/metabolic memory) within ulcers and in more distant tissues, that cause delayed healing and a chronic ulcerative phenotype, without the requirement of vascular and nerves insufficiency.
- Diabetes “damage memory” can be transmitted and impose the “damage phenotype message” in naïve recipient tissues. Thus, these “tissue priming factors” may be involved in the molecular pathogenesis of diabetic complications.
- T2DM ulcer granulation tissue, arteries, and nerves distant to the ulcer impaired healing recreated histological changes of angiopathy and neuropathy when injected into non-diabetic rat skin wounds, whereas normal human control tissues did not. Glycated bovine serum albumin delayed wound healing but did not reproduce vascular changes seen using T2DM tissues. Factors beyond glucotoxicity are involved in diabetics’ tissue damages.

## Data Availability Statement

The original contributions presented in the study are included in the article. Further inquiries can be directed to the corresponding author.

## Ethics Statement

The animal study was reviewed and approved by Dr. Jorge Castro-Velazco, president, Animal Welfare Board, CIGB, Animal Facility.

## Author Contributions 

JB-A contributed to study original idea and design; acquisition of data; analysis and interpretation of data; drafting of the manuscript; and studies supervision. MF-M contributed to study concept and design; acquisition and data curation; analysis and interpretation of data. YM-M contributed to study design; acquisition of data; analysis and interpretation of data. AG-O contributed to study design; acquisition of data; analysis and interpretation of data; drafting of the manuscript. GG-N contributed to study design, analysis and interpretation of data. RP contributed to analysis and interpretation of data and drafting of the manuscript. All authors contributed to the article and approved the submitted version.

## Funding

BioCubaFarma/IBM 3051-280 Research Project Account.

## Conflict of Interest

The authors declare that the research was conducted in the absence of any commercial or financial relationships that could be construed as a potential conflict of interest.

## Abbreviations

AGE, advanced glycation end products; BSA, bovine serum albumin; DM, diabetes mellitus; CFF, cells-free filtrate; IL-1β, interleukin 1β; IL-6, interleukin 6; MDA, malondialdehyde; RAGE, receptor for AGE; T2DM, type 2 diabetes mellitus; TNF-α, tumor necrosis factor-a; e-NOS, endothelial nitric oxide synthase; NF-κB, nuclear factor kappa-light-chain-enhancer of activated B cells; rh-EGF, recombinant human Epidermal Growth Factor.
